# Development of Cotton Picker Fire Monitoring System Based on GA-BP Algorithm

**DOI:** 10.3390/s23125553

**Published:** 2023-06-13

**Authors:** Weipeng Zhang, Bo Zhao, Shengbo Gao, Yuankun Zheng, Liming Zhou, Suchun Liu

**Affiliations:** 1 National Key Laboratory of Agricultural Equipment Technology, Chinese Academy of Agricultural Mechanization Sciences, Beijing 100083, China; zhwellpeng@gmail.com (W.Z.); gaoshengbo95@163.com (S.G.); caamsjds309@gmail.com (L.Z.);; 2 Department of Electrical and Mechanical Engineering, College of Engineering, China Agricultural University, Beijing 100089, China; uiagri.caams@gmail.com

**Keywords:** cotton picker, fire monitoring, GA optimized BP neural network, test verification

## Abstract

Due to the characteristics of the cotton picker working in the field and the physical characteristics of cotton, it is easy to burn during the operation, and it is difficult to be detected, monitored, and alarmed. In this study, a fire monitoring system of cotton pickers based on GA optimized BP neural network model was designed. By integrating the monitoring data of SHT21 temperature and humidity sensors and CO concentration monitoring sensors, the fire situation was predicted, and an industrial control host computer system was developed to monitor the CO gas concentration in real time and display it on the vehicle terminal. The BP neural network was optimized by using the GA genetic algorithm as the learning algorithm, and the data collected by the gas sensor were processed by the optimized network, which effectively improved the data accuracy of CO concentration during fires. In this system, the CO concentration in the cotton box of the cotton picker was validated, and the measured value of sensor was compared with the actual value, which verified the effectiveness of the optimized BP neural network model with GA. The experimental verification showed that the system monitoring error rate was 3.44%, the accurate early warning rate was over 96.5%, and the false alarm rate and the missed alarm rate were less than 3%. In this study, the fire of cotton pickers can be monitored in real time and an early warning can be made in time, and a new method was provided for accurate monitoring of fire in the field operation of cotton pickers.

## 1. Introduction

China is the largest cotton producer and consumer country in the world. By 2019, the cotton planting area exceeded 300 million hm^2^ and the output was about 5.889 million tons [1]. As the major cash crop and strategic material of the national economy, its planting area in Xinjiang is expanding continuously. Due to the labor shortage, cotton picker machinery became the main machinery for cotton harvesting. It can improve cotton picking efficiency, reduce labor costs, and increase economic benefits. However, the cotton picker machinery can easily cause a fire during the process of picking cotton. The main causes of fires are particularity of climate, terrain, as well as the physical characteristics of cotton, such as flammability and smoldering [2]. Therefore, the fire monitoring of cotton picker machinery is particularly important.

The main reason for fire in cotton pickers is the high concentration of inflammable gases in the chassis during the process of operation [3]. Therefore, the gas sensor is used to monitor the fluctuation of the concentration of inflammable gases in the cotton box of the cotton picker to reduce the fire rate [4]. A gas sensor can be used to monitor the CO concentration. The principle is that CO diffuses to the sensor and undergoes a reduction reaction [5] or oxidation reaction [6] on the catalytic electrode, and a current is generated on the working electrode [7]. The concentration of CO gas can be obtained by sampling and processing the current [8]. It is worth noting that high-precision gas monitoring sensor probes and strong sensor devices should be selected during the process of sensor selection [9]. Due to the accumulation of a large amount of cotton in the cotton box, the harvested cotton increases the difficulty of monitoring gas fluctuation with the ordinary sensor [10] and causes friction damage to the sensor [11]. The neural network is an operational model composed of a large number of connections between nodes [12]. It has an excellent ability to learn data features, so the prediction of fire situation can be realized by using the neural network to learn the data monitored by sensors [13]. One of the urgent problems to be solved is how to introduce the neural network algorithm into the fire monitoring of cotton picker and improve the monitoring performance [14]. Chen [15] integrated multi-modal sensors to obtain data of carbon monoxide, smoke, temperature, and humidity, and developed an indoor fire alarm system. The accurate fire warning information can be quickly obtained through the support vector machine algorithm. Sulistian [16] designed a fire early warning system, developed an alarm device, compared four machine learning classification methods, and determined that the classification algorithm can improve the performance of the intelligent fire alarm system.

It can be seen from the literature that the existing cotton pickers do not consider the problem of fire prevention, nor do they install relevant sensors on the cotton pickers. There is little research on fire prediction systems, and the fire prevention system of the cotton picker is very necessary. The optimized BP neural network model based on genetic algorithm was widely used in the prediction of agricultural machinery [17,18,19,20,21]. It has high accuracy and can be used for effective prediction. A fire monitoring system of a cotton picker based on the BP neural network model optimized by the GA genetic algorithm was designed.

In this paper, the CO concentration monitoring sensor and SHT21 temperature and humidity sensor were adopted to monitor the cotton box in real time. At the same time, the BP neural network was used to establish the prediction model of CO concentration, and the GA genetic algorithm was then used to accelerate the convergence of weight and threshold of BP neural network to optimize the prediction model. The prediction model of CO concentration can predict the probability of fire when the data collected by gas sensor were input into the prediction model. Finally, the automatic alarm and real-time monitoring can be achieved by transmitting the monitoring data of gas and the predicting data of fire to the on-board industrial control terminal system. Prior to this study, an intelligent detection and monitoring system for cotton bale storage based on FFID was designed, laying the foundation for this research.

## 2. Materials and Methods

### 2.1. Overall System Design

**Figure 1:**
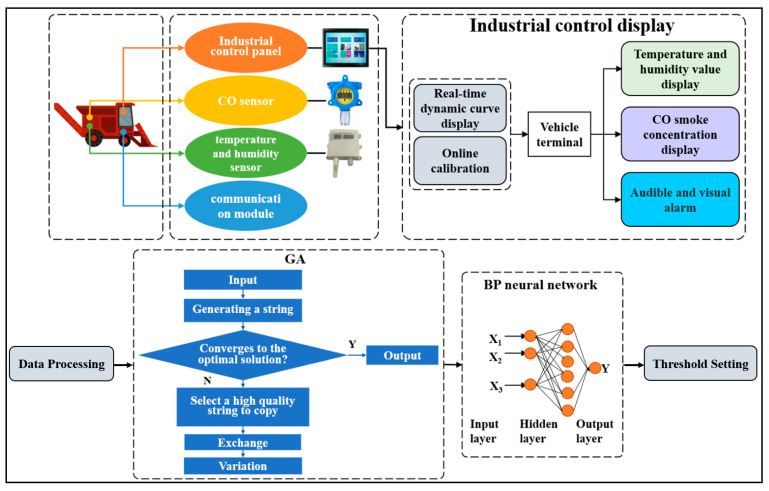
The overall structure of the fire monitoring system of the cotton picker.

The overall structure of the cotton picker fire monitoring system consists of three parts: a CO concentration sensor, a temperature and humidity sensor, and an on-board host computer. The CO concentration sensor mainly monitors the changes in CO concentration in the cotton box of the cotton picker. The temperature and humidity sensor mainly monitors the change of temperature and humidity in the cotton box to prevent smoldering. The data collected by the sensor were transmitted to the host computer system through RS485 communication serial port and stored. Then, the stored data were trained by GA-BP model to obtain an optimized model. As a display unit, the on-board terminal can display the sensor data in real time, and can set thresholds to realize automatic alarm and other functions. The overall structure of the cotton picker fire monitoring system is shown in Figure 1. When a fire occurs, the CO sensor will detect the corresponding gas concentration. When smoldering occurs, the temperature and humidity sensor will detect the changes in temperature and humidity inside the box. Then, the industrial control screen will display the curves of co concentration, temperature, and humidity. Then, the GA-BP algorithm will predict the threshold of gas concentration, temperature, and humidity based on the data collected by the sensor. Finally, according to the predicted values, the upper computer in the cab sets off the sound and light alarm to complete the fire monitoring and warning function.

### 2.2. Hardware System Design

**Figure 2:**
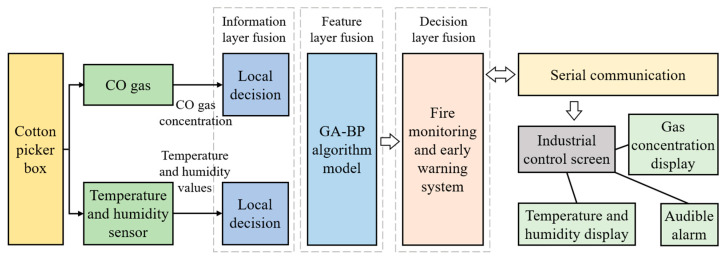
Hardware system structure diagram.

The hardware of cotton picker fire monitoring and early warning system includes a CO gas monitoring module, a temperature and humidity monitoring module, and a industrial control host computer. The structure of hardware system is shown in Figure 2.

#### 2.2.1. CO Gas Monitoring Module

**Figure 3:**
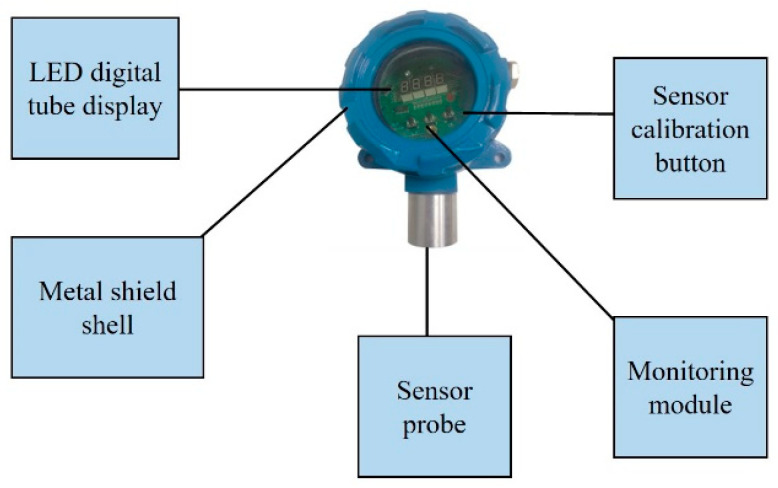
Structure block diagram of CO gas monitoring sensor.

In order to effectively improve the monitoring accuracy of the gas sensor, a high-precision gas concentration monitoring sensor was adopted in CO gas concentration monitoring module, with working temperature: −30 °C~+50 °C, working humidity: ≤90%RH, DC24V ± 15%. The sensor had an LED digital tube display function, the CO gas measurement range was 0–1000 ppm, the resolution was 1 ppm, and the response time ≤20 s. The sensor can monitor CO with high precision, wide range, short response time, and more accurate monitoring. In addition, the output mode was a three-wire system, with a current signal range 4~20 mA and the output signal of RS485 isolation. The incomplete combustion of flames will generate CO with a large amount of smoke, so the monitoring module needs to measure the fluctuation of CO concentration in the air in real time. The gas sensor included a digital tube display, real-time calibration, high-precision probe, and metal shell, as shown in Figure 3. The sensor was powered by 2 24 V power supply, and the change of potential of output terminal was directly related to the change of the CO gas concentration.

#### 2.2.2. Temperature and Humidity Module

**Figure 4:**
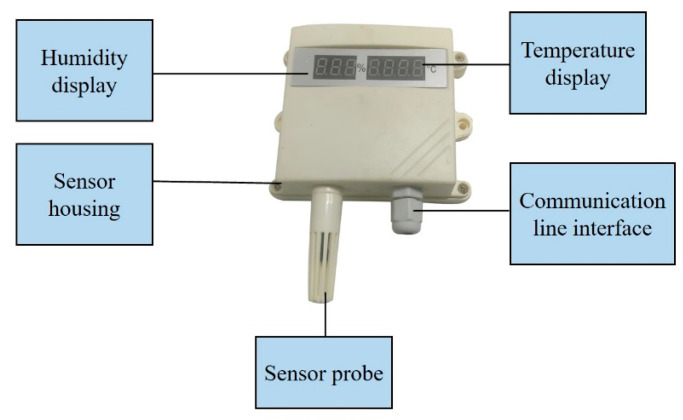
Structure block diagram of temperature and humidity monitoring sensor.

The SHT21 temperature and humidity sensor was selected for the temperature and humidity detection module. The temperature range of the sensor was: −30~70 °C (±0.2 °C) and the humidity range of the sensor was 0~100%RH (±3%RH). The response time was <1 s, temperature of working environment temperature was −55 °C~120 °C (±0.1 °C), and the humidity of working environment was ≤95%RH (±0.1%RH). The RS485 with standard Modbus-RTU protocol was adopted for communication serial port, with the device default address 01. The temperature and humidity module had a sensor probe, temperature and humidity digital display, sensor housing, and communication interface, as shown in Figure 4. Among them, the sensor probe had the function of high-precision information acquisition. It can display temperature and humidity changes in real time, and can convert the received temperature variable signals into standardized output signal and input them into the industrial control computer after processing. The advantages of the sensor probe are fast response speed and high accuracy.

#### 2.2.3. Host Computer Display Module

**Figure 5:**
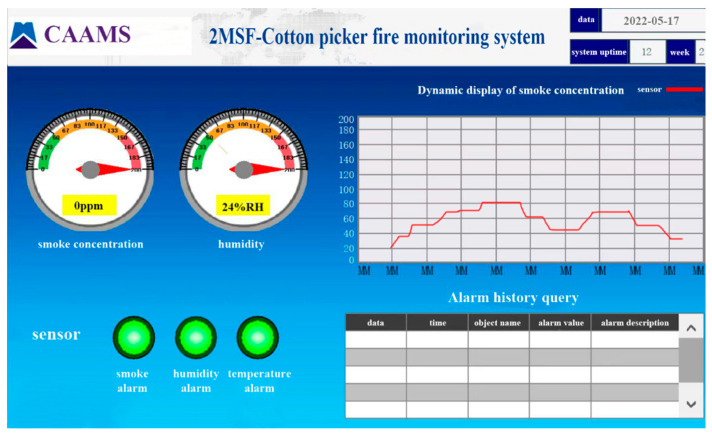
Host computer system.

The MCGS touch screen (Kunlun Technology, Shenzhen, China) used as the human–computer interaction system module of the upper computer monitoring terminal had a 4-core (main frequency 1 GHz) CPU as the core. Additionally, the communication interface included 1*RS232; 2*RS485; 1*LAN network port, built-in MCGS professional version, four-wire resistive touch screen, TPC1031Ki, resolution 1024 × 600, supported a serial port multi-protocol communication, and can communicate directly with the CO sensor, as well as the temperature and humidity sensor. As shown in Figure 5, the upper computer display included communication status information, the temperature and humidity of the cotton box of the cotton-picking machine, and the CO gas concentration value. The upper computer had an alarm indicator light, etc., which can display the change of the fire warning situation in the cotton box in real time, and the observation was simple and convenient.

### 2.3. Software System Design

#### 2.3.1. Host Computer System Design

**Figure 6:**
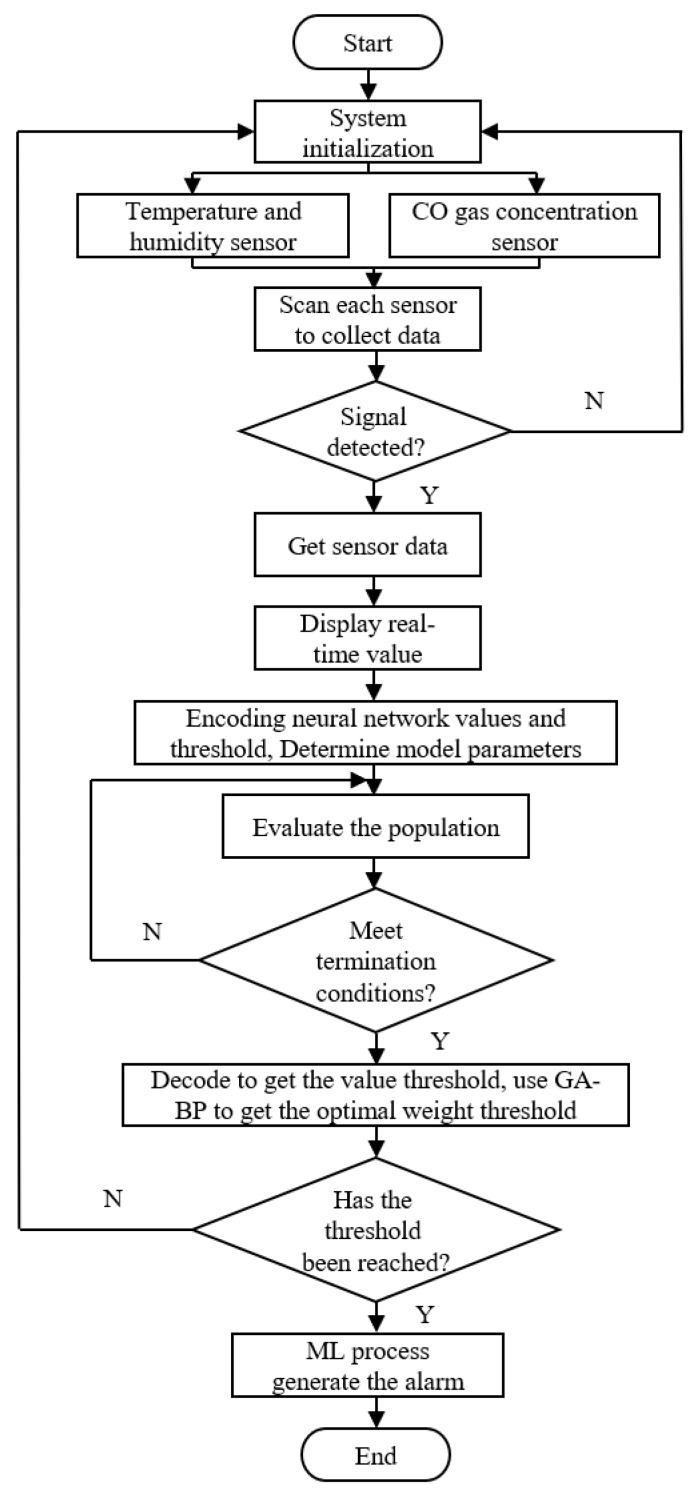
Fire warning system software flow chart.

In the design, the collection of temperature and humidity, CO concentration of the sensing module, logical judgment and alarm, data transmission, and display of monitoring data were realized. Firstly, the system was initialized, including RS485 communication rate and mode setting, temperature and humidity sensor, CO concentration sensor and alarm threshold setting, etc., and the prescription chart was loaded. The initial alarm threshold was 50 ppm, but the threshold value would be affected by the environment, and the algorithm would be used to predict it later. Then, the data receiving timer was started, and the information of CO sensor and temperature and humidity sensor was received and processed through the RS485 communication line. At the same time, the dynamic concentration change curve was obtained to complete the fire warning. The software program flow is shown in Figure 6.

#### 2.3.2. System Algorithm Design

(1) GA-BP neural network definition

The BP back propagation algorithm is a well-known method for training multilayer feedforward artificial neural networks. By adding gradually trained data and hidden units, the learning convergence speed can be accelerated to avoid falling into local minimum. The GA algorithm is a method to search the optimal solution by simulating the natural evolution process. The GA-optimized BP algorithm model can improve the global optimization ability of traditional GA algorithm and BP algorithm, respectively, and improve the prediction efficiency and accuracy of the algorithm. Therefore, through the CO concentration and temperature and humidity information uploaded by the gas sensor, the GA genetic algorithm was used to train the weights and thresholds of the fire monitoring and early warning neural network, which can avoid the problem of falling into the local optimal solution due to the use of the traditional BP neural network, and can improve the accuracy of fire monitoring and early warning.

(2) GA-BP neural network model construction

**Figure 7:**
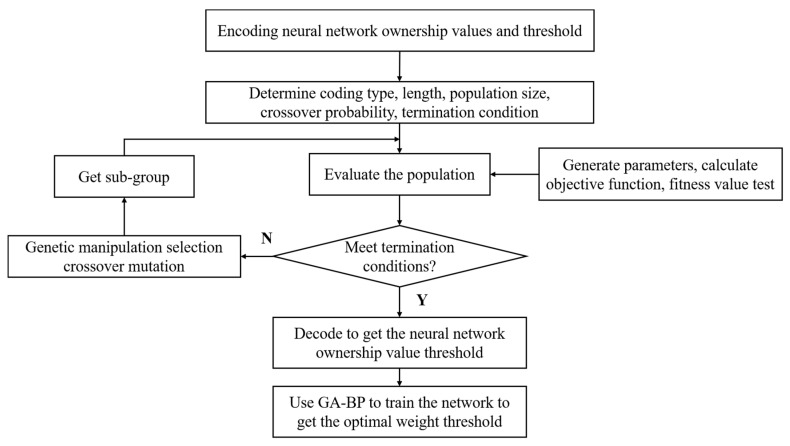
Algorithm block diagram of GA to optimize the weights of BP neural network.

As shown in Figure 7, the specific algorithm process of optimizing the neural network connection weight based on genetic algorithm was as follows:

(1) A set of distributions are randomly generated through the information of CO concentration, temperature, and humidity uploaded by the gas sensor, and each weight in the group is encoded by a certain coding scheme, so as to construct a code chain. Under the premise that the network structure and learning algorithm have been determined, the code chain corresponds to the neural network with specific weights and thresholds.
(2) The error function of the generated neural network is calculated to determine its fitness function value, and the error is inversely proportional to the fitness.
(3) The individuals are sorted by the method of fitness ratio, and a number of individuals with larger fitness values are selected and inherited directly to the next generation.
(4) Crossover operation is performed, and two individuals are randomly selected from the population to exchange with each other according to the set probability.
(5) The mutation operation is carried out, random mutation points are defined, and improved genetic operators are used to form a new generation of groups through adaptive adjustment of individuals such as crossover and mutation.
(6) Steps (2) to (5) are repeated to make a set of initially determined weight distribution evolve continuously until the training target is satisfied or the number of iterations reaches the preset target.

In this paper, the traditional BP feed forward neural network model, the IPSO optimized BP neural network model, and the GA-BP neural network model were trained, the training results were obtained, and the best neural network model was selected to carry out the fire prediction analysis of the cotton picker. The model capabilities were compared by NSE, MAE, RMSE, and the formula was as follows:

(1) $$NSE=1-\frac{\sum_{i=1}^{n}{\left(X_{i\rho }-X_{i\xi }\right)}^{2}}{\sum_{i=1}^{n}{\left(X_{i\rho }-{\bar{X}}_{0}\right)}^{2}}$$

(2) $$MAE=\frac{\sum_{i=1}^{n}\left|X_{i\rho }-X_{i\xi }\right|}{n}$$

(3) $$RMSE=\sqrt{\frac{\sum_{i=1}^{n}{\left(X_{i\rho }-X_{i\xi }\right)}^{2}}{n}}$$

where $X_{i\rho }$ is Actual value uploaded by gas sensor. $X_{i\xi }$ is predicted value by GA-BP algorithm. ${\bar{X}}_{0}$ is arithmetic mean of the actual values uploaded by the gas sensor. n is number of data points.

### 2.4. Test Verification

**Figure 8:**
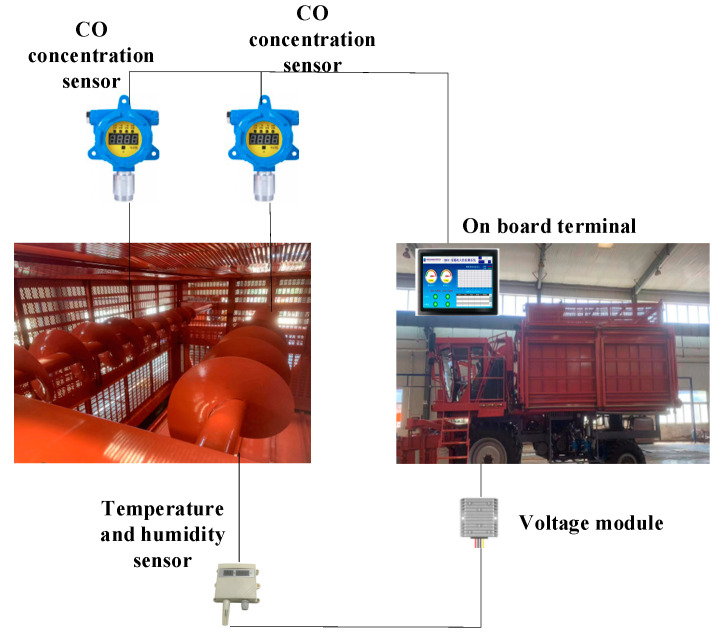
Test site photos.

The model cotton picking box was taken as the research object. As shown in Figure 8, the length, width, and height of the box were 3 × 2 × 2 m. The CO sensor and temperature and humidity sensor were fixed and installed in the cotton box, and the display screen of the industrial computer was connected to the cab. During the test, the cotton was lit in the cotton box of the cotton picker, the state of the cotton fire was tested during the whole process, the changes of the characteristic parameters were observed, and the warning effect was recorded. The system was able to operate continuously for a long time and meet the operation requirements of cotton pickers.

## 3. Results

### Algorithm Performance Evaluation

Since the numerical dimensions and ranges of CO concentration, temperature, and humidity were all different, in order to make reasonable use of these three characteristic variables, normalization was performed. The normalization formula is

(4) $$Y_{i}^{\prime }=\frac{Y_{i}-Y_{\min}}{Y_{\max}-Y_{\min}}$$

where $Y_{i}$ is Current input data. $Y_{\min}$ is the smallest value in the data. $Y_{\max}$ is the largest value in the data. $Y_{i}^{\prime }$ is Normalized value.

**Figure 9:**
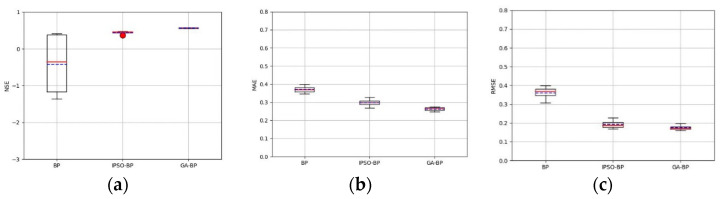
Performance comparison of different algorithms for (**a**) NSE (Nash efficiency coefficient), (**b**) MAE (mean absolute error), and (**c**) RMSE (root mean square error).

CO concentration was taken as an example in this paper to evaluate the performance of the algorithm. The parameters of CO concentration were input into three BP neural networks. Additionally, one of them was optimized by IPSO (Improved Particle Swarm Optimization), and the other by GA (Genetic Algorithm). In order to truly reflect the difference of prediction results among the three models, the three models were trained 20 times, respectively, in this paper, and statistical analysis was performed on the NSE (Nash efficiency coefficient) and MAE (mean absolute error), and the RMSE (root mean square error) was calculated for each prediction, as shown in Figure 9.

The greater the difference between the boxes in the box plot, the greater the difference in the evaluation indicators of the model prediction results. It can be seen from Figure 9a that there was a significant overall difference in the evaluation indicators of the prediction results between the BP neural network model and the GA-BP neural network model. Additionally, there was a significant overall difference in the evaluation indicators of the prediction results between the IPSO-optimized BP neural network model and the GA-BP neural network model. The GA-BP box in Figure 9b,c was the smallest, and the GA-BP neural network model had the smallest variability. The smaller the MAE and RMSE, the more stable the algorithm. The values of MAE and RMSE of the GA-BP neural network model were slightly smaller than those of the BP neural network model and the IPSO-optimized BP neural network model, and the NSE was slightly higher than the BP neural network model and the IPSO-optimized BP neural network model. The network model showed that GA-BP neural network can effectively improve the stability and accuracy of prediction.

**Figure 10:**
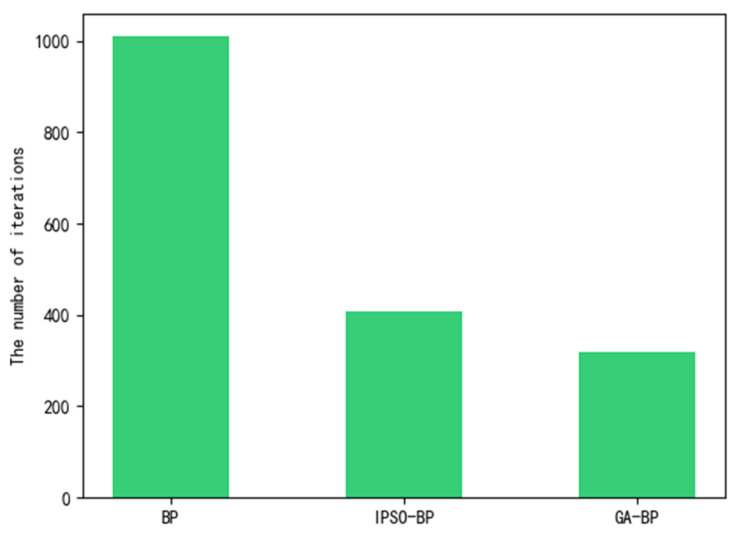
Comparison of iteration times of different algorithms.

The total number of iterations of the BP neural network model optimized by the IPSO particle swarm algorithm, the BP neural network model optimized by the GA genetic algorithm, and the traditional BP model were statistically analyzed, as shown in Figure 10.

**Figure 11:**
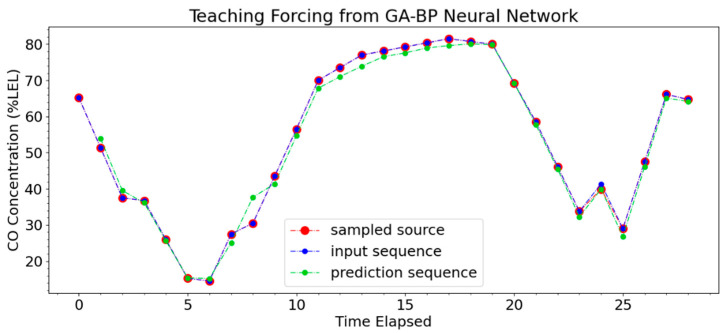
GA-BP neural network model prediction.

The number of iterations of the GA-BP neural network model in Figure 10 was less than that of the BP neural network model and the BP neural network model optimized by IPSO, indicating that the GA-BP neural network can reduce the number of iterations. The fewer iterations, the greater the memory consumption caused by the experiment and the lower calculation cost. Therefore, the GA-BP model was selected in this paper to predict the fire situation of the cotton picker. The CO concentration parameters uploaded by the gas sensor were uploaded to the trained GA-BP neural network model, and the output value of the fire prediction time was obtained. The prediction accuracy curve is shown in Figure 11.

It can be seen from Figure 11 that the predicted curve had a high coincidence rate with the actual curve, with an error rate less than 3%, and an accuracy rate of 96.84%. The GA-BP neural network model can effectively predict and warn the fire situation, which greatly increased the safety.

## 4. Discussion

### 4.1. System Monitoring Effect Comparison

In order to verify the effect of prediction algorithm, the GA-BP algorithm was compared with the GA algorithm and the IPSO-BP algorithm. The temperature, humidity, and co concentration were monitored 100 times by the sensor, and the average value of the actual value was compared with the average value of the predicted value. The value measured by KT-600 gas detector (Henan Baoshian Co., Ltd., Zhengzhou, China) and Xima AS847 industrial grade temperature and humidity meter (Dongguan Wanchuang Electronic Products Co., Ltd., Dongguan, China) was taken as the real value, and the value judged by adding the sensor to the algorithm was selected as the monitoring value. The results are shown in Table 1.

The average error was obtained by comparing the humidity and CO concentration error comparison, and the error used by the system was 3.44%. The fire monitoring and early warning algorithm of the cotton picker based on GA-BP designed in this study was more accurate than the other two algorithms. The reason for this was that in the algorithm, the GA genetic algorithm was used to replace the traditional method of randomly generating weights and thresholds, and the adjustment of the BP neural network error back propagation was used to optimize the weights and thresholds gradients to reduce errors, avoid falling into the local minimum value, obtain the optimal network structure, and make the model more robust. The error curve of the predicted value of the algorithm in this paper was in good agreement with the experimental measurement value, and the error was small, which also reflected the systematic and scientific nature of cotton picker operation safety. The GA-BP algorithm was adopted in the early warning and monitoring system of the cotton picker to realize the fire early warning model of the cotton picker, so as to obtain better fire warning effects.

### 4.2. System Alarm Performance Comparison

The performance of early warning system is an important indicator to measure the fire early warning effect of the cotton picker. The fire alarm performance will affect the safety of the cotton picker to a large extent. Therefore, in order to enhance the interpretability of the experimental results, the effectiveness of the algorithm can be effectively highlighted by taking the fire early warning results as the index in this paper. BP and IPSO-BP algorithms were compared and the performance verification was completed together with the algorithm in this paper, as shown in Table 2.

It can be seen from the table that the fire warning algorithm based on the BP neural network had a recognition accuracy of 91.57% in 700 fire recognitions. The BP algorithm fire warning based on IPSO optimization had an accurate recognition rate of 93.14% in 700 fire identifications. The BP fire warning algorithm based on GA optimization had an accurate recognition rate of 96.86% in 700 fire identifications. Through comparison, it can be seen that the accuracy of GA-BP algorithm was higher than that of BP neural network algorithm and IPSO-BP algorithm. The GA-BP algorithm was superior to the other two methods.

## 5. Conclusions

Taking the CO concentration value, temperature, and humidity of the cotton box of the cotton picker as the fire monitoring object, the gas concentration and temperature and humidity changes in the cotton box of the cotton picker were monitored in real time by using the CO sensor and temperature and humidity sensor. Combined with the GA-optimized BP neural network intelligent algorithm, the accurate monitoring system of CO concentration in cotton box realized the functions of gas concentration data collection and storage, data real-time transmission of RS485, real-time display of vehicle-mounted industrial computer and alarming when data exceeded the threshold. The system was convenient, simple, and clearly displayed, and had a good human–computer interaction interface.

In view of the problems of high monitoring sensitivity during cotton pickers fires, sensor damage caused by long-term extrusion, and low accuracy of data in cotton box due to interference of gas sensor by field environment, high-precision gas detection sensor probes, and metal shell sealed sensor were adopted, and the data measured by the gas sensor were optimized by the BP network model optimized by the GA genetic algorithm, which effectively improved the data accuracy. The CO gas concentration was used as model input for the experiment. The experimental results showed that the monitoring system worked well and realized the function of system design. At the same time, after BP neural network model was optimized by the GA genetic algorithm, the measurement accuracy of CO was not less than 91.23%, which can effectively improve the monitoring accuracy of early warning of cotton box fire. In order to ensure reliable data transmission, the RS485 transmission mode was adopted in the system.

## 6. Limitations and Future Research

Because the existing cotton pickers do not consider fire protection, there is no sensor installed in the cotton picker. There is little research on fire prediction systems, and so, the fire protection system of cotton picker is very necessary. The sensors used in the study were not fully functional and in the future, data transmission can be carried out through mature wireless equipment. At the same time, fire-fighting and rain-spraying devices can be installed for fire monitoring. It can be seen from the test and detection that the system has the potential to be applied to a cotton picker fire warning, and the system will be further improved in the future.

## Figures and Tables

**Table 1:** Comparison of monitoring effects of different algorithms.

Algorithm	Times	Temperature (Average Value)	Humidity (Average Value)	CO Concentration (Average Value)	Mean Relative Error
Actual value	100	32.7	50.6	35.1	-
BP	100	33	48.3	32.9	3.91%
IPSO-BP	100	33.2	44.6	32	7.40%
GA-BP	100	32.9	47.7	33.7	3.44%

Table 1 shows that in the comparison process of the three algorithms, by calculating the temperature.

**Table 2:** Comparison of fire warning algorithms.

Algorithm	Test Number	Accurate Recognition Times/n	Accuracy
BP	700	641	91.57%
IPSO-BP	700	652	93.14%
GA-BP	700	678	96.86%

## Data Availability

Not applicable.
